# Flexible Fiber Probe for Efficient Neural Stimulation and Detection

**DOI:** 10.1002/advs.202001410

**Published:** 2020-06-09

**Authors:** Minghui Du, Lu Huang, Jiajun Zheng, Yue Xi, Yi Dai, Weida Zhang, Wei Yan, Guangming Tao, Jianrong Qiu, Kwok‐Fai So, Chaoran Ren, Shifeng Zhou

**Affiliations:** ^1^ State Key Laboratory of Luminescent Materials and Devices School of Materials Science and Engineering South China University of Technology Guangzhou 510640 China; ^2^ Guangdong Provincial Key Laboratory of Fibre Laser Materials and Applied Techniques Guangdong Engineering Technology Research and Development Center of Special Optical Fibre Materials and Devices Guangzhou 510640 China; ^3^ Guangdong‐Hongkong‐Macau Institute of CNS Regeneration Ministry of Education CNS Regeneration Collaborative Joint Laboratory Jinan University Guangzhou 510632 China; ^4^ Department of Neurology and Stroke Center The First Affiliated Hospital of Jinan University Guangzhou 510632 China; ^5^ Research Laboratory of Electronics Massachusetts Institute of Technology (MIT) Cambridge MA 02139 USA; ^6^ School of Optical and Electronic Information Wuhan National Laboratory for Optoelectronics Huazhong University of Science and Technology Wuhan 430074 China; ^7^ College of Optical Science and Engineering State Key Laboratory of Modern Optical Instrumentation Zhejiang University Hangzhou 310027 China; ^8^ Guangzhou Regenerative Medicine and Health Guangdong Laboratory Guangzhou 510530 China; ^9^ Co‐innovation Center of Neuroregeneration Nantong University Nantong 226001 China; ^10^ Center for Brain Science and Brain‐Inspired Intelligence Guangdong‐Hong Kong‐Macao Greater Bay Area Guangzhou 510000 China

**Keywords:** brain–machine interfaces, flexible fiber probes, neural recording, neural stimulation, optogenetics

## Abstract

Functional probes are a leading contender for the recognition and manipulation of nervous behavior and are characterized by substantial scientific and technological potential. Despite the recent development of functional neural probes, a flexible biocompatible probe unit that allows for long‐term simultaneous stimulation and signaling is still an important task. Here, a category of flexible tiny multimaterial fiber probes (<0.3 g) is described in which the metal electrodes are regularly embedded inside a biocompatible polymer fiber with a double‐clad optical waveguide by thermal drawing. Significantly, this arrangement enables great improvement in mechanical properties, achieves high optical transmission (>90%), and effectively minimizes the impedance (by up to one order of magnitude) of the probe. This ability allows to realize long‐term (at least 10 weeks) simultaneous optical stimulation and neural recording at the single‐cell level in behaving mice with signal‐to‐noise ratio (SNR = 30 dB) that is more than 6 times that of the benchmark probe such as an all‐polymer fiber.

## Graphical abstract

A category of tiny fiber probes with metal electrodes regularly embedded inside the double‐clad biocompatible polymer fiber is realized. This arrangement enables excellent mechanical properties, high transmission (>90%), and low impedance (22.71 MΩ cm^2^ at 1 kHz), which can realize long‐term simultaneous optical stimulation and neural recording at the single‐cell level in behaving mice with high signal‐to‐noise ratio.

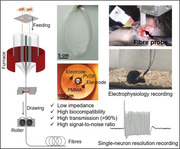

Optogenetics, which involves the manipulation of light to control molecular events in a desired manner in living tissues,^[^ ^1^ ^]^ present promising applications in neural regeneration,^[^ ^2^ ^]^ brain disorder diagnostics (e.g., Parkinson's disease, Alzheimer's disease, and depressive disorder),^[^ ^3^, ^4^, ^5^, ^6^, ^7^, ^8^ ^]^ gene editing,^[^ ^9^ ^]^ tumor immunology,^[^ ^10^ ^]^ artificial intelligence,^[^ ^11^, ^12^ ^]^ protein coding, and cell manipulation technologies.^[^ ^13^, ^14^, ^15^, ^16^, ^17^ ^]^ The success of optogenetics greatly benefits from the invention of neuron probes, which bridges the connection between the complex nervous system and the external world. Various probing devices for optogenetics, based on silicon microelectrode arrays,^[^ ^18^, ^19^ ^]^ neuropixels,^[^ ^20^, ^21^ ^]^ neurogrids,^[^ ^22^, ^23^ ^]^ mesh electronics,^[^ ^24^, ^25^, ^26^, ^27^ ^]^ dense 3D silicon probe,^[^ ^28^, ^29^ ^]^ silicon probe integrated with micro‐LEDs and polymer electrode arrays,^[^ ^30^, ^31^ ^]^ have been reported. These probes have demonstrated the powerful capability for detection of the electrophysiological signal, while the mechanical properties of these probes, including bending stiffness, storage modulus etc., do not match with the soft neural tissues.^[^ ^32^ ^]^ Moreover, these probes are mostly limited in long‐term neural signal recording due to the decrease of signal‐to‐noise ratio (SNR) and the inflammatory response between the probe and the tissues. Further extension of its function for simultaneous stimulation and signaling is greatly limited and increasing attention is focused on this task because of its technological relevance with the smart nerve‐machine interface.^[^ ^32^, ^33^, ^34^, ^35^, ^36^, ^37^ ^]^ Therefore, it is interesting to develop multifunctional tiny probes with excellent mechanical properties, high biocompatibility, excellent optical transmission, and low impedance, etc., and explore their potential applications for long‐term simultaneous stimulation and signaling.

Here, we have developed a flexible multimodal fiber probe that is constructed based on a combined scale‐down and constant‐scale codrawing strategy. The fabricated flexible multimodal fiber probe is composed of metal electrode used for neural signal recording, and a double‐clad waveguide for optical stimulation. To improve the optical transmission property, a double‐clad waveguide is employed to confine the light in the inner core, and the metal electrode is embedded in the outer layer of the fiber probe. This configuration gives the fiber probe excellent optical transmission property (>90%), high flexible and mechanical properties, and lower specific impedance (22.71 MΩ cm^2^ at 1 kHz). Moreover, the functional fiber probe can be produced in high throughput by thermal drawing technology with lower cost. We further demonstrate the long‐term simultaneous optical stimulation and neural recording of the fiber probe at the single cell level in behaving mice with significantly high signal‐to‐noise ratio (SNR = 30 dB).

To construct the desired multimodal fiber probe, we propose a combined constant‐scale and scale‐down codrawing strategy. This drawing means that the size remains constant or exhibits continuous change during fiber drawing. As illustrated in **Figure** **1**a, they were performed in parallel while being controlled by independent controllers #1 and #2. This process enables integration of multimaterials, which are totally incompatible through the conventional fabrication method. As a proof‐of‐concept model, the multimodal fiber probe with metal electrodes as recording electrode and double‐clad waveguide as stimulating tunnel was constructed. The fabrication procedures were summarized as follows: On the one hand, the double‐clad rod was designed by selecting polycarbonate (PC) as core and polyvinylidene fluoride (PVDF) as clad because their notable contrast in refractive index (*n*_PC_ = 1.59 and *n*_PVDF_ = 1.42) helps to generate a waveguide that facilitates highly efficient light transmission. They were inserted into a protective polymethyl methacrylate (PMMA) tube, and the final PC–PVDF–PMMA composite rod was used for further scale‐down drawing. It was monitored by sensor #2 and dynamically tuned by controller #2, until the target size and configuration were achieved. On the other hand, the metal electrodes (e.g., platinum, gold, and copper) was fed through the hollow channels pre‐designed in the PC–PVDF–PMMA composite rod with the help of sensor #1 and controller #1. The diameter of the metal electrodes remains constant during the entire drawing process due to its melting point is much higher than the fiber drawing temperature (280 °C). Furthermore, the quantities and positions of recording electrodes can be easily tuned by rational design of the macroscopical preform.

**Figure 1:**
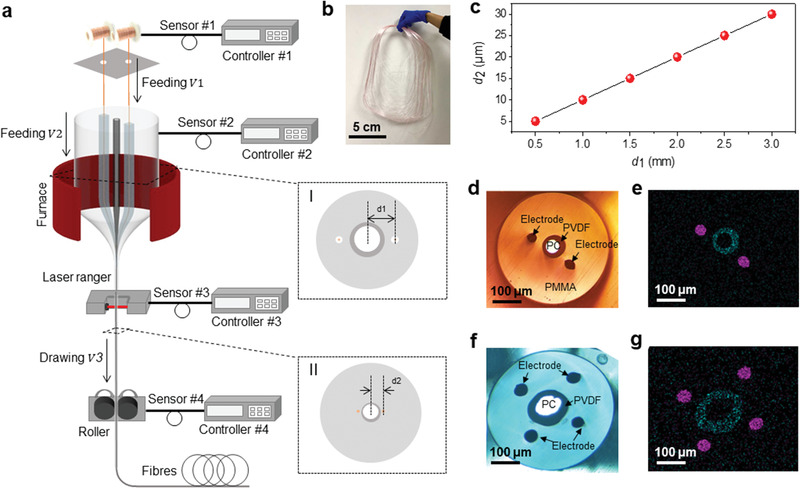
Construction of multimodal fiber probe. a) Illustration of combined constant‐scale and scale‐down co‐drawing strategy. The metallic filaments and preform are fed inside the furnace with feeding speed of *V*_1_ and *V*_2_, which is tuned via controllers #1 and #2, respectively. Insets I and II illustrate the change of cross‐section from multimaterial preform to multimodal fiber. Except for metallic filaments, the scale of other structural units is gradually reduced during codrawing. The size of the final product is determined by the ratio of the capstan and feeding speeds, which is continuously monitored by a laser sensor. b) A photograph of the constructed product. c) The linear dependence of the characteristic scale of the fiber probe on the predesigned configuration as illustrated in the inset of (a) after fixing the drawing parameters (e.g., size and speed). d–g) Optical micrographs and corresponding element mapping for two typical configurations with the waveguide surrounded by two (d,e) and four electrodes (f,g), respectively.

The aforementioned strategy exhibits several notable advantages. One of the attractive features is that it is fully compatible with the commercial fiber‐drawing technique and can produce multimodal fibers with highly uniform size, configuration and high yields.^[^ ^38^ ^]^ In principle, multimodal fiber probes that are more than 100 m long can be obtained per hour and a bundle of fabricated product is exhibited in Figure 1b. Another prominent advantage involves the ability to rationally control the separation distance between the electrode and waveguide. As illustrated in Figure 1a, the characteristic size of the fiber is gradually reduced in the neck‐down region during fiber drawing. Thus, we can anticipate that after fixing the feeding and drawing parameter (*V*_1_, *V*_2_, and *V*_3_), the separation distance *d*_2_ is scaled linearly with the initial *d*_1_. This relationship was directly confirmed by six individual tests, and samples with *d*_2_ = 5, 10, 15, 20, 25, and 30 µm can be effectively obtained (Figure 1c). Furthermore, the organization manner of the electrode and waveguide can be conveniently tuned (Figure 1d–g and Figure S1, Supporting Information). Figure 1d–g exhibits two typical configurations with the waveguide surrounded by two and four electrodes, respectively. The corresponding element mapping indicates the isolated electrical and optical functional units and double‐clad geometry of the central waveguide.

The fabricated multimodal fiber probe is highly flexible, as demonstrated in **Figure** **2**a, which shows it wrapping around a small PC rod with diameter of ≈300 µm. The dynamic mechanical properties of the probes were evaluated by characterizing the bending stiffness, stress and storage modulus in the frequency range of mammalian locomotion, respiration and heartbeat (0.01–10 Hz). Figure 2b shows the frequency‐dependent bending stiffness of the multimodal fiber and commercial probes with different diameters range from 150 to 300 µm. The bending stiffness of the multimodal fiber probe with the diameter of 200 µm is ≈350 N m^−1^ with the frequency range of 0.01–10 Hz, which is ≈10 times lower than that of the commercial silica probe (≈3500 N m^−1^) with the comparable dimensions. The bending stress of the multimodal fiber probe is estimated to be about 12.5 ± 1.4 MPa, which is substantially smaller than that of the commercial probe (125 ± 1.3 MPa) (Figure 2c). Results demonstrate the high flexibility and excellent bending resistance of the constructed multimodal fiber probes. Moreover, the storage modulus of the multimodal fiber probe (50.5 ± 4.2 GPa) is also much lower than that of the commercial probe (580.3 ± 5.1 GPa) (Figure 2d), indicating its great restoration capacity after bending deformation. Moreover, the mechanical properties of the fiber probe can be improved by the rational design of the fiber diameter. The robust mechanical properties of the multimodal fiber probe imply that it can potentially experience compressive or tensile stress,^[^ ^39^ ^]^ thereby it would be an ideal candidate as next‐generation neural probe for in vivo applications.

**Figure 2:**
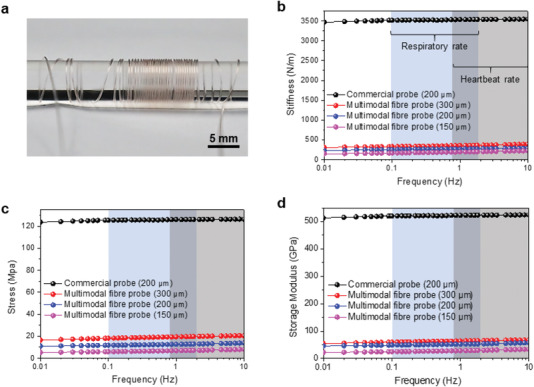
Mechanical characteristics of multimodal fiber probe. a) A photograph of the drawn multimodal fiber probe wrapped around a PC rod. b) Graph showing the measured bending stiffness for the multimodal fiber probe and commercial silica probe at various frequencies and diameters, including those of respiration and heartbeat across different mammals. The displacement amplitude was 50 µm. c) Bending stress of multimodal fiber probe and commercial silica probe. d) Storage modulus of multimodal fiber probe and commercial silica probe.

The double‐clad configuration of the optical functional unit gives the multimodal fiber probe excellent light guide properties. The optical transmission spectra and the corresponding image indicate that they exhibit high transmission (>90%) in a wide waveband range (400–800 nm) (**Figure** **3**a and Figure S2, Supporting Information). The stability of the multimodal fiber probe was tested by soaking it in phosphate buffered solution (PBS, pH = 7.4) or cerebrospinal fluid (CSF) solution for 10 or 30 days, respectively. The corresponding transmission spectra show negligible changes, confirming the super stability of the multimodal fiber probe (Figure 3a). As optogenetic experiments often involve channelrhodopsin 2 (ChR2), we further quantified the optical transmission along the multimodal fiber probe at the ChR2 characteristic peak (at 473 nm) (Figure 3b). The optical loss is fitted to be ≈1.18 ± 0.04 dB cm^−1^, which is lower than that of commonly used polymer probes, such as SU‐8 (6.4 dB cm^−1^).^[^ ^40^ ^]^

**Figure 3:**
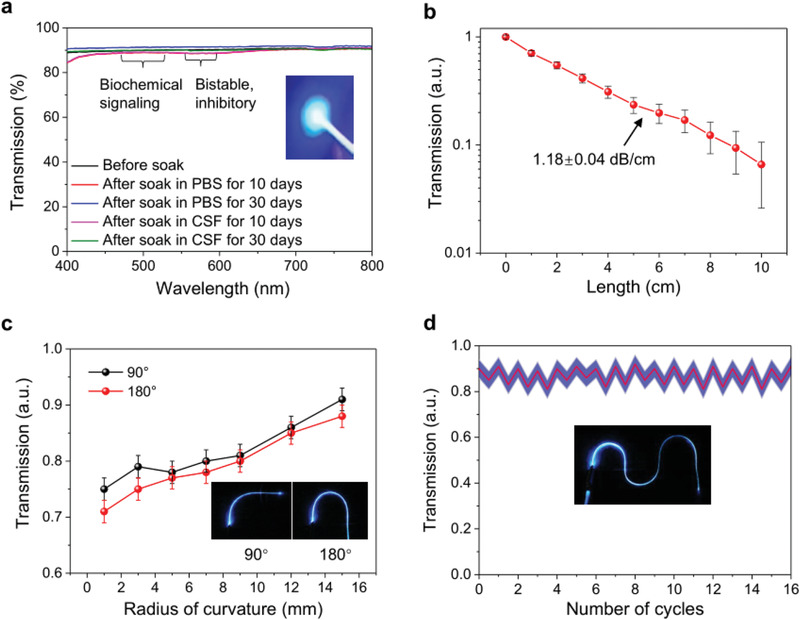
Optical characteristics of multimodal fiber probe. a) Transmission spectra of multimodal fiber probe in the visible range (400–800 nm) and fiber probe being soaked in PBS or CSF solution for 10 and 30 days, respectively. The inset is a photograph showing the 473 nm blue light transmitted through the fiber probe. b) Transmission of multimodal fiber probe as a function of the fiber length ranges from 0 to 10 cm. The measured optical loss (dB cm^−1^) of the fiber probe is 1.18 ± 0.04 dB cm^−1^. c) Optical transmission through multimodal fiber probes at 90° and 180° deformation with radii of curvature of 0.5–15 mm. The inset photograph shows the transmission of 473 nm blue light through the multimodal fiber probes at 90° and 180° deformation. d) Transmission was measured in the relaxed and deformed 180° angle and radius of curvature = 3 mm (16 repetitions). No decrease in performance was found. The inset is a photograph showing the transmission of 473 nm blue light through the multimodal fiber probe at several 180° deformations.

Deformation loss of the multimodal fiber probe is another critical parameter for in vivo application because it frequently occurs during movement of the living body. To evaluate deformation loss, we measured the radius‐of‐curvature dependent optical transmission at 473 nm at the deformation angles of 90° and 180° (Figure 3c). Approximately 72% transmission can be retained at 180° with 1 mm radius of curvature, which is superior to the commercial silica fiber probes. The fatigue degradation was also measured under repeated strain during locomotion. A typical 16‐cycle test with 180° deformation at 3 mm radius of curvature was performed and no appreciable decline in performance can be observed (Figure 3d). Thus, the observed optical loss during bending deformation process can be attributed to the optical scattering in the fiber rather than the damage of the fiber structure.^[^ ^41^ ^]^

The third major advantage of the fabricated multimodal fiber probe is the electrical recording function provided by the pure platinum, gold or copper filaments (purity = 99.99%) which is homogeneously distributed along the axial direction of the fiber (**Figure** **4**a,b). To study it, the impedance of the multimodal fiber probe was recorded using an electrochemical impedance spectrum (EIS). Benefiting from its metallic feature, the corresponding impedance of the multimodal fiber probe shows a significant decrease in the frequency range from 10^2^ to 10^4^ Hz, and estimated to be 22.71 MΩ cm^2^ at the frequency of 1 kHz with one electrode (Figure 4c and Figure S5 in the Supporting Information), which is critically important for achieving efficient electrophysiological recording with single‐spike resolution. Moreover, the impedance of the fiber probe can be tuned by rational design of the diameter and the number of the metal electrodes. The electrical conduction performance of the multimodal fiber probe was also compared with the other reported neural probes made of Ag nanowire‐polymer composites,^[^ ^34^ ^]^ CPE conductive polymer,^[^ ^41^ ^]^ graphene fiber composites,^[^ ^42^ ^]^ and carbon nanotube‐polymer composites.^[^ ^43^ ^]^ Significantly, the impedance of multimodal fiber probe is about one order lower than that of the reported polymer probes, demonstrating its promise for highly precise electrical detection and recording. The measured average phase angle at 1 kHz is about −67.3° (Figure 4d and Figure S5 in the Supporting Information), indicating that a capacitive charging‐discharging process, and it is not the irreversible faradaic reactions, controls the electrochemical interaction at the exposed tip of the multimodal fiber probe. This would decrease the signal to noise ratio (SNR) of recording neural signals.^[^ ^42^, ^43^ ^]^ However, this can avoid the risk of tissue damage from irreversible faradaic reactions based on early works.^[^ ^44^ ^]^

**Figure 4:**
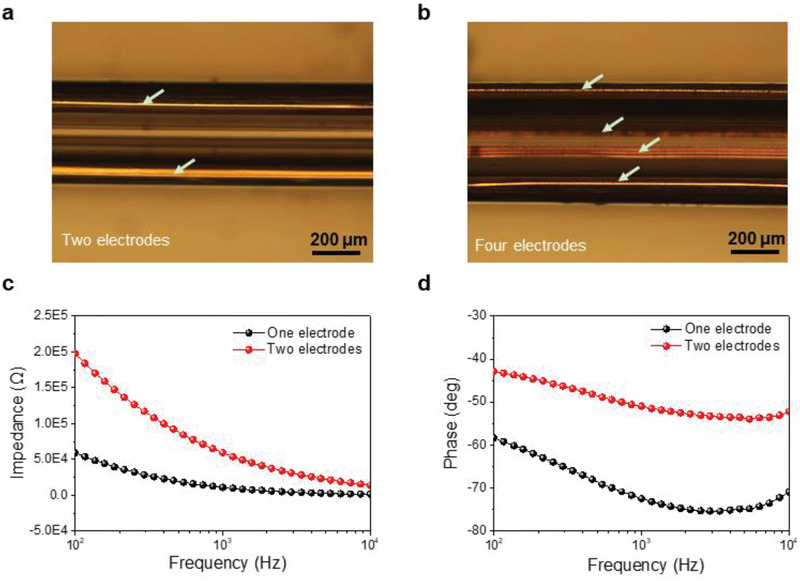
Electrical properties of multimodal fiber probe. a,b) Reflection optical images of fiber probe showing that two and four electrodes are homogeneously distributed along the axial direction of the fiber. c,d) The Bode magnitude and phase of impedance of various electrodes in multimodal fiber probe, respectively.

The success in construction of the novel multimodal fiber probe with tiny size (<0.3 g, Figure S3, Supporting Information) and attractive properties prompted us to further explore the possibility to record and manipulate neural activity in behaving mice that are expressing opsins. We injected the superior colliculus (SC) of the mice with AAV containing a channelrhodopsin‐2 (ChR2)‐mCherry fusion protein, which has been shown to evoke action potential during in vivo recordings.^[^ ^45^, ^46^ ^]^ Three weeks later, the multimodal fiber probe with two electrodes (diameter of 50 µm) was implanted into the SC of the mice (**Figure** **5**a,b and Figure S4, Supporting Information). The electrodes in the multimodal fiber probe were combined for single channel electrophysiology recording, and the exposed surface area of the recording electrodes was estimated to be ≈1963.5 µm^2^. Robust ChR2 expression in the SC was observed three weeks after the injection, and the extent of expression was proportional to the injection volume (Figure 5c). The mice were placed in the open field for 10 min and exposed to 5 ms pulses of illumination (*λ* = 473 nm, continuous light, power density of 10 mW mm^−1^ at the tip of the implanted fiber, 9.94 mW mm^−1^ at 0.5 mm and 9.88 mW mm^−1^ at 1 mm below the fiber). Significantly, neural stimulation and electrophysiological signaling can be simultaneously achieved by a single multimodal fiber probe (Figure 5d,e). The SNR is calculated to be more than 6 times higher than the reported probes,^[^ ^42^, ^43^ ^]^ which mainly benefits from the excellent electrical properties confirmed in Figure 4. Moreover, the signal can be detected at any arbitrary time point and only a slight decrease of SNR (≈20%) occurs after 10 weeks (Figure 5e–h). The amplitude of the recording neural signal keep stability at the frequency of 1 to 20 Hz after 10 weeks of implantation. Above results demonstrate its long‐term functionality and stability in freely moving mice.

**Figure 5:**
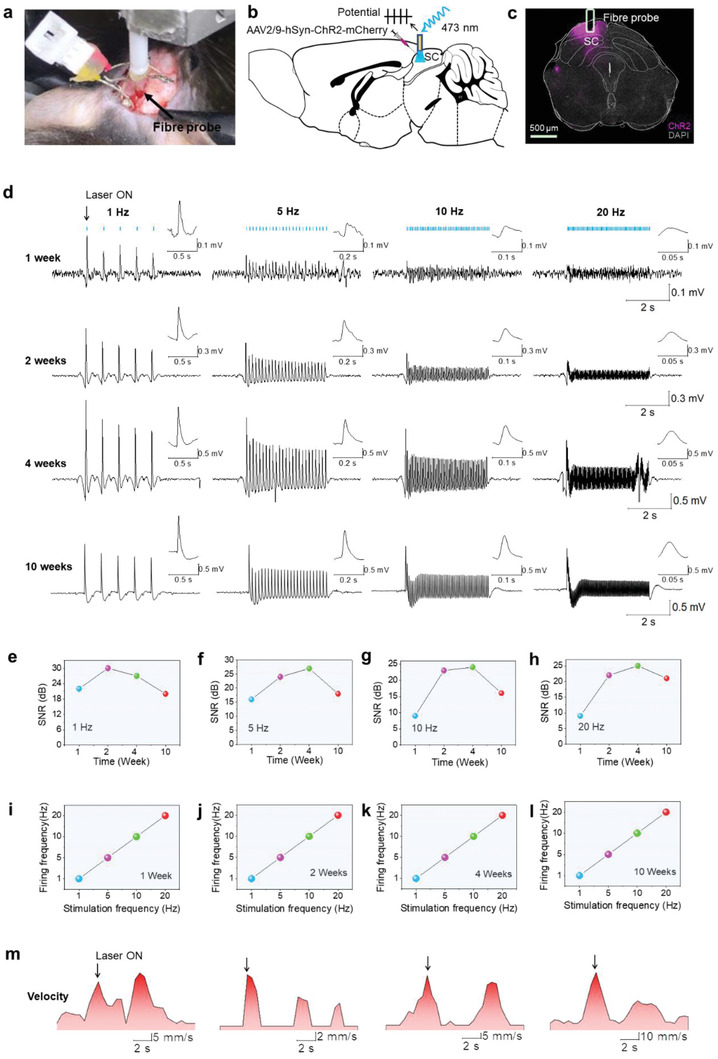
Simultaneous optogenetic stimulation and electrophysiological recording of multimodal fiber probe in vivo. a) Photographic image of a mouse implanted with multimodal fiber probe. b) Schematic of multimodal fiber implanted into the SC. c) Confocal image of a coronal section across the SC three weeks after viral transfection. d) Simultaneous optogenetic stimulation (5 ms pulse width, 1–20 Hz; blue marks indicate laser onsets) and electrophysiological recording was performed from 1 week to 10 weeks after implantation. Action potentials isolated from the recording was also performed in the top right corner. e–h) SNR of recorded potential from 1 week to 10 weeks under different frequency stimulations. i–l) The relation between the firing frequency and optical stimulation frequency for SC neuron at 1 week, 2, 4, and 10 weeks, respectively. m) Optical stimulation dependent movement velocity of the behaving mice after implantation with multimodal fiber probes for 4 weeks.

We further analyzed the characteristics of the spikes, such as the amplitude, the amplitude area, the rising and decay time of action potentials, isolated from the recording potentials from 1 week to 10 weeks in the stimulation frequency range of 1 to 20 Hz (Figure S6 in the Supporting Information). Because the expression of ChR2 usually takes at least two weeks,^[^ ^47^ ^]^ the average amplitude and area of the spikes at 1 week is relatively lower than 2, 4, and 10 weeks. The average amplitude and area of the spikes decreases with the increasing of frequency from 1 to 20 Hz, and the highest average peak and area was recorded at 4 weeks. After 10 weeks of implantation, a high peak amplitude of about 2.2 mV can still be obtained by the multimodal fiber probe. The rising time of the spikes shows no noticeable change by the increase of implantation time and frequency, while the decay time decreases significantly with the increase of frequency. The decrease of amplitude, area and decay time with the increase of frequency may be caused by the differences of neuronal subtypes, network connection modes, and the amount of light received by neurons.^[^ ^32^, ^45^ ^]^ More interestingly, we observed the bandwidth of the spike signal remains constant at different recording time points, indicating its origin from the single neural unit. This finding provides a new opportunity to study the online light‐cell interaction with high time and space resolution. For example, we investigated the relation between the stimulation frequency and electrophysiological response behavior, which has been recognized as a key parameter in optogenetics.^[^ ^48^ ^]^ As shown in Figure 5d, the neural behavior in a frequency‐dependent manner (from 1 to 20 Hz) can be clearly observed at any time point. On the one hand, the firing and stimulation frequency exhibit a perfect linear relationship that confirms the reliability of the multimodal fiber probe and stimulability over a wide frequency range (Figure 5i–l). On the other hand, with the frequency increasing from 1 to 20 Hz, the signals are distinguished from each other, indicating the tunable light‐nerve interaction.

Finally, we demonstrate the success in manipulating the behavior of the mice. The SC contributes to defensive behavior such as freezing.^[^ ^49^ ^]^ We found that the defensive mode with increased freezing of active mice can be effectively triggered upon photo stimulation (Movie S1, Supporting Information), which is further revealed by the stimulation dependent movement velocity (Figure 5m). The results collaboratively demonstrate that the multimodal fiber probe allows for simultaneous optogenetic stimulation and electrophysiological recording with high resolution and sensitivity, pointing to promising applications in brain–machine interface.

The implantation of neural probe for long periods can induce the formation of glia scar, which encapsulates the probe and insulates the interface of the probe.^[^ ^50^ ^]^ To determine the biocompatibility of our newly developed multimodal fiber probe, we compared the tissue response to our newly developed multimodal fiber probe and commercial silica probe by immunohistochemical analysis of brain slices across the implantation site at 4 different time points after implantation (1 week, 2, 4, and 10 weeks). As illustrated in **Figure** **6**a, brain slices were counterstained with calcium‐binding adaptor molecule 1 (Iba1), which is a marker of the activated microglia.^[^ ^51^ ^]^ Cell density and cell area of the SC Iba1^+^ SC neurons located in the implantation site were further analyzed (Figure 6b,c). For the commercial probe and our newly developed multimodal fiber probe, increases in the cell area and cell density of Iba1^+^ SC neurons were observed from 1 week to 10 weeks after implantation (Figure 6a–c). However, compared with the commercial probe group, our multimodal fiber probe group showed that the cell area of the Iba1^+^ SC neurons was significantly smaller at 2, 4, and 10 weeks after implantation (Figure 6a,b), and the cell density of Iba1^+^ SC neurons in the multimodal fiber probe group was significantly lower at 2 weeks after implantation (Figure 6a,c). Those results suggest that our newly developed multimodal fiber probe has better biocompatibility than the commercial probe.

**Figure 6:**
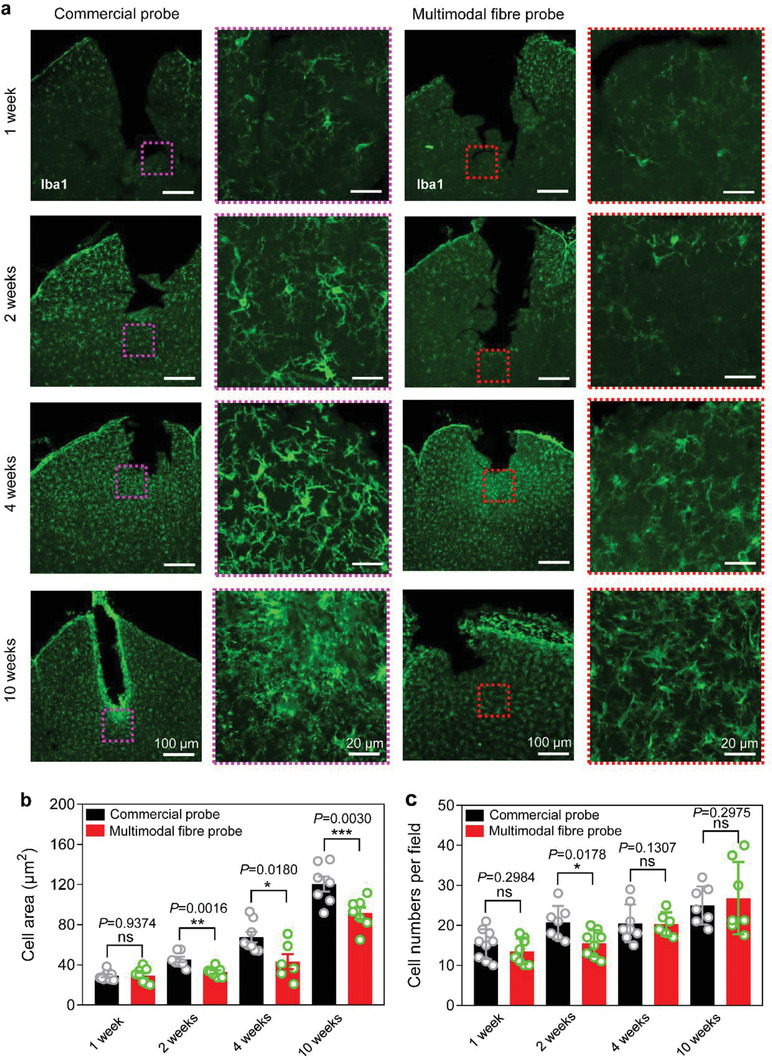
Evaluation of tissue response of multimodal fiber probes. a) Representative confocal images of SC illustrating the distribution of the Iba1^+^ SC neurons (green) in the implantation site 1 week, 2, 4, and 10 weeks after fiber implantation. b,c) Statistical analysis of cell area and cell density of Iba1+ SC neurons in different groups. Data presented as mean ± s.e.m.

In conclusion, we have demonstrated a novel biocompatible multimodal fiber probe with attractive properties, including small size, great flexibility, excellent optical transmission and extremely low electrical impedance. This combination enables long‐term simultaneous optical stimulation and neural recording with high resolution and sensitivity. We demonstrated the stimulation‐feedback function at the single cell level in freely moving mice with a new record of SNR value. This multimodal fiber probe is believed to create a new nerve–machine interface, and therefore may revolutionize the fields of neuroscience, biomedicine, healthcare, intelligent robotics, disorder diagnostics, and artificial intelligence. Furthermore, the works may also stimulate new concepts on the next‐generation fiber probe derived from the multimaterials.

## Experimental Section

##### Fabrication of Multimodal Fiber Probe

To fabricate multimodal probes, PC, PVDF, and PMMA rods were selected as raw materials for core, inner clad, and outer clad. In a typical synthesis procedure, a PMMA rod with size of 30 mm in diameter and 12 cm in length was selected, and a hole with diameter of 8 mm and length of 10 cm was machined inside the rod. Similarly, a PVDF rod with size of 8 mm in diameter and 10 cm in length was selected, and a hole with diameter of 3 mm and length of 10 cm was machined inside it. Then, a PC rod with diameter of 3 mm and length of 10 cm was inserted into the PVDF tube, and the PC–PVDF rod was inserted into the PMMA tube to obtain the PC–PVDF–PMMA preform. The PC–PVDF–PMMA preform was then consolidated at 180 °C for 1 h. After consolidation, the preform was machined with two holes with the designed size of 2 mm in diameter and 12 cm in length. The preform was then transferred to a modified fiber‐drawing tower for fabrication of the fiber probe. It was constructed based on the classic thermal drawing process in a three‐zone heating furnace, where the top, middle and bottom zones were heated to 160, 280, and 120 °C, respectively. As illustrated in Figure 2, the preform was fed into the furnace at a rate of 1 mm min^−1^ and drawn at a speed of 2 m min^−1^, resulting in a draw‐down ratio of ≈100. The tungsten or copper filaments with diameter of 50 µm were continuously fed into the preform during the draw. The entire fiber‐drawing process was carefully and dynamically tuned via controllers #1 and #2 until the highly homogeneous fiber was obtained. More than 100 m of fiber can be collected from each draw per hour.

##### Microstructure Characterization

The optical images and cross sections of the multimodal fiber probes were captured by a Leica MDI5000 microscope (Germany). The SEM images and mapping of the elements were obtained using a Merlin SEM (30 kV, BSE Zeiss, Germany).

##### Optical Properties

The optical transmission spectra were measured using a Perkin–Elmer Lambda 900 UV/VIS/NIR spectrophotometer (Waltham, MA, USA). To determine the optical transmission loss, a 473 nm laser light source was selected as the probe beam and was coupled into the fiber. The light output intensity with different fiber lengths was measured with a photodetector that was attached to a power meter (PD 300‐UV, 200–1100 nm, OPHIR). To test the optical guiding properties of the multimodal fiber probe during bending, one end of the fiber was fixed and the other end was attached to a moving stage. The radius‐of‐curvature dependent optical transmission was measured by varying the distance between two ends.

##### Mechanical Properties

The bending stiffness was characterized on a dynamic mechanical analyzer (DMA, Q800, TA Instruments). The samples with length of 1.5 cm were mounted with a single cantilever clamp. The samples were then tested with a frequency sweep (0.01–10 Hz) under controlled displacement (50 µm) at 37 °C.

##### Electrochemical Impedance Spectrum (EIS)

One end of the multimodal fiber probe was dissolved to release the electrodes. Measurements were performed using an electrochemical workstation (Interface 1000, Gamry) with a sinusoidal input (10 mV, 10 Hz–10 kHz), adopting three‐electrode in the phosphate buffered saline (PBS) solution. The fiber probe, Ag/AgCl and Pt wire were employed as the working electrode, the reference electrode and the counter electrode, respectively.

##### Surgery and Intracranial Injection

All experiments were approved by the Jinan University Institutional Animal Care and Use Committee. Adult male (6–8 weeks old) C57BL/6 mice were used in this study. Animals were housed under a 12 h:12 h light‐dark cycle (lights on at 7 AM) with food and water provided ad libitum. The mice were anesthetized (Avertin, 13 µL g^−1^, i. p.) and placed in a stereotaxic instrument (RWD, Shenzhen, China). Erythromycin eye ointment was applied to prevent corneal drying and a heat pad was used to hold body temperature at 37 °C. A small craniotomy hole was made using a dental drill (OmniDrill35, WPI) and a micropipette connected to a nanoliter injector (Nanoliter 2010, WPI, Sarasota, FL) and its controller (Micro4; WPI, Sarasota, USA) was used for injection. To specifically infect SC neurons with ChR2‐mCherry, mCherry, AAV2/9‐hSyn‐ChR2‐mCherry or AAV2/9‐hSyn‐mCherry was injected into the SC of C57BL/6 mice (virus titers: 3–5 × 1012 particles mL^−1^; 0.15 mL per injection); AP: −3.7 mm; ML: −0.55 mm and DV: −1.2 mm).

##### Injection Site Verification

After transcardial perfusion with 0.9% saline followed by 4% PFA (paraformaldehyde) in 0.1 m PBS (phosphate‐buffered saline), the brain was removed and post‐fixed with 4% paraformaldehyde overnight at 4 °C, and then transferred into 30% sucrose until sectioning with a cryostat (CM1900, Leica Microsystems, Bannockburn, USA). A series of 50 mm sections were collected for verification of injection sites; tissue was examined under epifluorescence using a Zeiss Axioimager Z2 microscope.

##### Immunocytochemistry

All animals were anaesthetized (Avertin, 13 µL g^−1^, i.p.) and perfused intracardially with 0.9% saline followed by 4% PFA in 0.1 m PBS. Brains were removed for detection of Iba1, and then were isolated and washed in 0.1 m PBS three times (10 min each) before incubation in 0.1 m PBS containing 10% normal goat serum (Vector Laboratories, Burlingame, CA, USA) and 0.3% Trition‐X‐100 (T8787, Sigma‐Aldrich, St. Louis, MO, USA) for 1 h. Then, the brains were incubated for 2 days at 4 °C with the Iba1 antibody (rabbit polyclonal antibody 019–19741, 1:500, Wako Pure Chemicals Industries, Osaka, Japan,). This step was followed by six rinses in 0.1 m PBS and then incubation with a secondary (Dylight 488) goat‐anti‐rabbit IgG (1:400, Vector Laboratories) for 6 h at room temperature.

##### Image Analysis

Brains and sections were imaged with a Zeiss 700 confocal microscope with 5 × or 20 × objectives. Each stack of optical sections covered a retinal area of 325.75 × 325.75 mm^2^ (1024 × 1024 pixels). Using Image J and Photoshop CS6 (Adobe Corp., San Jose, California, USA), each stack was montaged of optical sections and projected it to a 0° *X*–*Y* plane and a 90° *Y*–*Z* plane to obtain a 3D reconstruction of the cell. Contrast and brightness were adjusted. For each Iba1 labeled neuron, the total sizes of the soma and dendritic field were analyzed. Dendritic field area was calculated by drawing a convex polygon linking the dendritic terminals. The dendritic field area was then measured, and the diameter was calculated as that of a circle having an equal area.

## Conflict of Interest

The authors declare no conflict of interest.

## Supporting information

Supporting Information

Supplemental Video 1
