# Supplementary material for: Flexible Fiber Probe for Efficient Neural Stimulation and Detection
Source: Adv Sci (Weinh). 2020 Jun 9;7(15):2001410. doi: 10.1002/advs.202001410 (PMC7404151; doi:10.1002/advs.202001410)
Supplement: Supplementary file 1 — Supporting Information [file ADVS-7-2001410-s001.pdf]

© 2020 Wiley-VCH GmbH

ADVANCED SCIENCE
Open Access

Supporting Information

for *Adv. Sci.*, DOI: 10.1002/advs.202001410

Flexible Fiber Probe for Efficient Neural Stimulation and Detection

Minghui Du, Lu Huang, Jiajun Zheng, Yue Xi, Yi Dai, Weida Zhang, Wei Yan, Guangming Tao, Jianrong Qiu, Kwok-Fai So, Chaoran Ren, and Shifeng Zhou**

Copyright WILEY-VCH Verlag GmbH & Co. KGaA, 69469 Weinheim, Germany, 2020.

Supporting Information

Flexible fibre probe for efficient neural stimulation and detection

Minghui Du[1,2,11], *Lu Huang*[3,4,11], *Jiajun Zheng*[4,11], *Yue Xi*[4], *Yi Dai*[1,2], *Weida Zhang*[1,2], *Chaoran Ren*[4,5,6,7]*, *Wei Yan*[8], *Guangming Tao*[9], *Jianrong Qiu*[10], *Kwok-Fai So*[4] & *Shifeng Zhou*[1,2]*

[1]State Key Laboratory of Luminescent Materials and Devices, School of Materials Science and Engineering, South China University of Technology, Guangzhou 510640, China

[2]Guangdong Provincial Key Laboratory of Fibre Laser Materials and Applied Techniques, Guangdong Engineering Technology Research and Development Center of Special Optical Fibre Materials and Devices, Guangzhou 510640, China

[3]Department of Neurology and Stroke Center, The First Affiliated Hospital of Jinan University, Guangzhou 510632, China

[4]Guangdong-Hongkong-Macau Institute of CNS Regeneration, Ministry of Education CNS Regeneration Collaborative Joint Laboratory, Jinan University, Guangzhou 510632, China

[5]Guangzhou Regenerative Medicine and Health Guangdong Laboratory, Guangzhou 510530, China

[6]Co-innovation Center of Neuroregeneration, Nantong University, Nantong 226001, China

[7]Center for Brain Science and Brain-Inspired Intelligence, Guangdong-Hong Kong-Macao Greater Bay Area, Guangzhou 510000, China

[8]Research Laboratory of Electronics, Massachusetts Institute of Technology (MIT), Cambridge, MA 02139, USA

[9]School of Optical and Electronic Information, Wuhan National Laboratory for Optoelectronics, Huazhong University of Science and Technology, Wuhan 430074, China

[10]College of Optical Science and Engineering, State Key Laboratory of Modern Optical Instrumentation, Zhejiang University, Hangzhou 310027, China

[11]These authors contributed equally to this work.

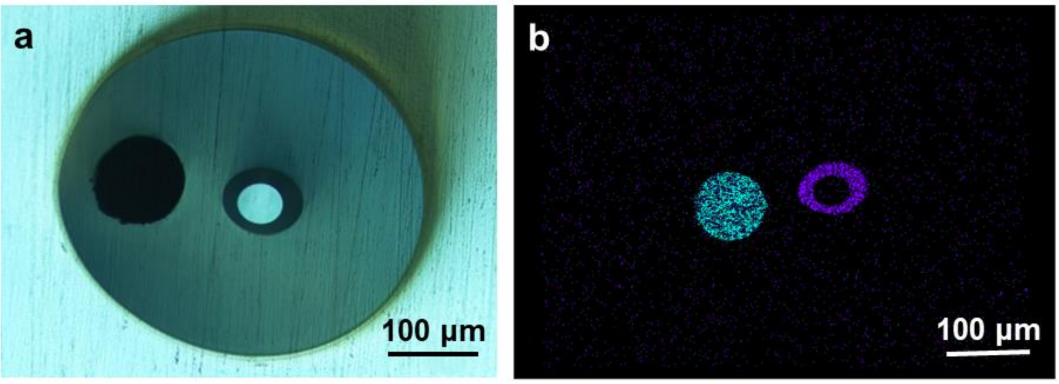

Figure S1. Optical micrograph a) and scanning electron microscopy (SEM) mapping b) of the cross-section of multimodal fibre probe, showing one metallic electrode embedded in the fibre probe.

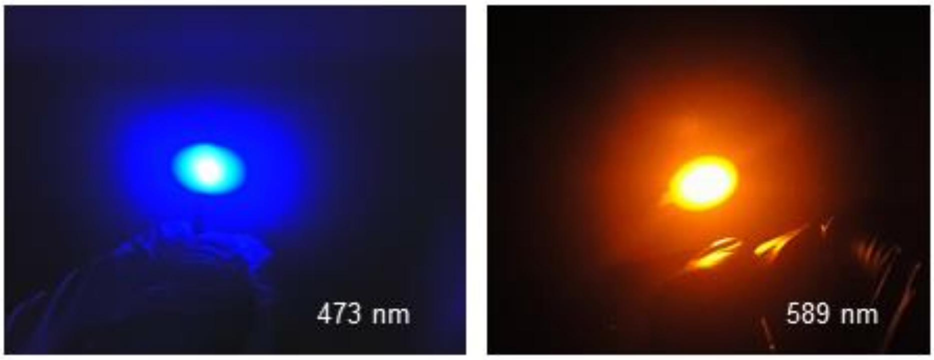

Figure S2. Digital photograph of the light (λ = 473 nm and 589 nm, respectively) coupled into the multimodal fibre probe.

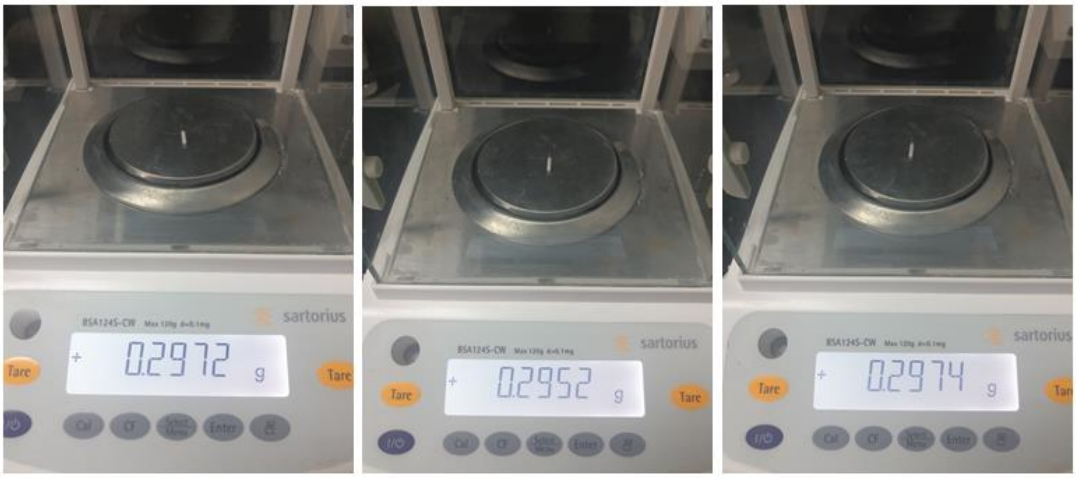

Figure S3. The weight of three multimodal fibre probes solidified into a ceramic ferrule using optical epoxy. The results show that the total weight of the multimodal fibre probe and the ceramic ferrule is less than 0.3 g.

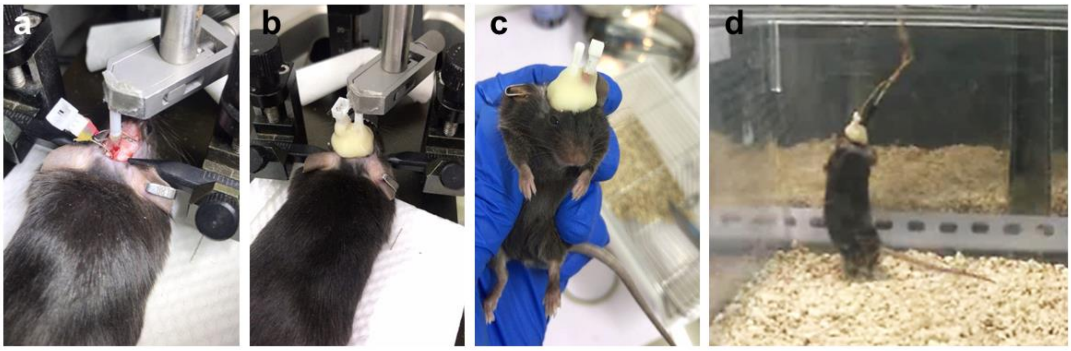

Figure S4. a-d) The implantation of multimodal fibre probe into SC of the brain of the mice for *in vivo* electrophysiology experiment.

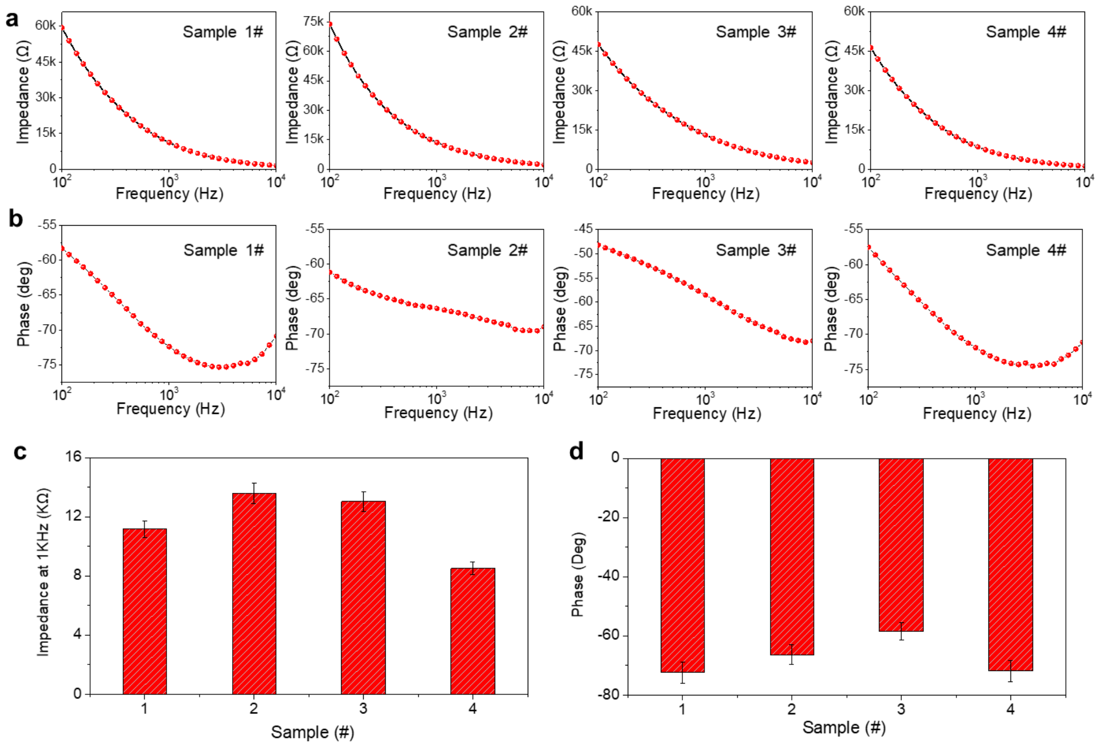

Figure S5. (a, b) The measured impedance and phase of four fibre probes. (c, b) The statistics results of impedance and phase at 1 kHz.

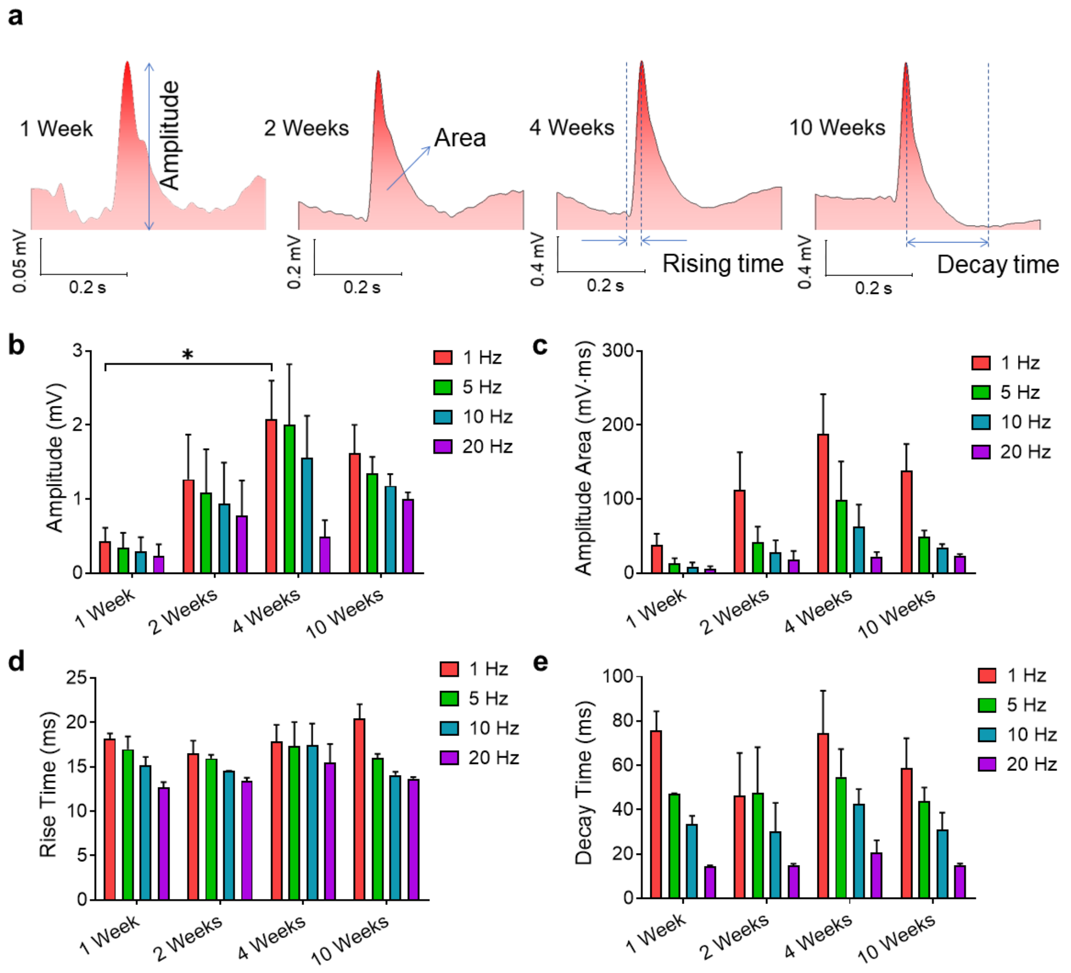

Figure S6. The characteristics of the spikes. (a) The schematic diagram of amplitude, amplitude area, rise time and decay time of a single spike isolated from the recordings in 1 week, 2, 4 and 10 weeks, respectively. (b-e) The statistics results of average amplitude, amplitude area, rising time and decay time of action potentials from 1 week to 10 weeks after implantation in the stimulation frequency range of 1 Hz to 20 Hz.

Table S1. Physical and chemical properties of the materials employed for fabrication of the multimodal fibre probe

Materials	Heat deflection temperature (°C)	Tensile modulus (GPa)	Young's modulus (GPa)	Refractive index
COC	170	3200	3	1.53
COP	138	2900	3	1.51
PC	150	2300	2.38	1.59
ABS	180~200	2000	2.20	1.54
PVDF	132	1380	2	1.42
PMMA	130~135	2100~2400	3	1.58
PS	70~100	3200	3~3.60	1.60
Silica	1667	-	69	1.46

Supplementary References

1. Mark, J.E. Polymer data handbook. (Oxford University Press, New York, USA, 1999).
2. Biron, M. Thermoplastic and thermoplastic polymers. (William Andrew, Kidlington, UK, 2013).
3. Wypych, G. Handbook of Polymers. (ChemTec Publishing, Toronto, Canada, 2012).
4. Hull, R. Properties of Crystalline Silicon. (The Institution of Electrical Engineers, London, UK, 1999).
